# Genotypic Characterization of *Escherichia coli* O157:H7 Isolates from Different Sources in the North-West Province, South Africa, Using Enterobacterial Repetitive Intergenic Consensus PCR Analysis

**DOI:** 10.3390/ijms15069735

**Published:** 2014-05-30

**Authors:** Collins Njie Ateba, Moses Mbewe

**Affiliations:** 1 Department of Biological Sciences, North West University, Mafikeng Campus, Private Bag X2046, Mmabatho 2735, South Africa; 2 Department of Water and Sanitation, University of Limpopo, Turfloop Campus, Private Bag X1106, Sovenga 0727, South Africa; E-Mail: moses.mbewe@ul.ac.za

**Keywords:** *E. coli* O157:H7, enterobacterial repetitive intergenic consensus (ERIC) sequences, bacterial source tracking (BST), genetic fingerprints, unweighted pair group method with arithmetic mean

## Abstract

In many developing countries, proper hygiene is not strictly implemented when animals are slaughtered and meat products become contaminated. Contaminated meat may contain *Escherichia coli* (*E.* *coli*) O157:H7 that could cause diseases in humans if these food products are consumed undercooked. In the present study, a total of 94 confirmed *E. coli* O157:H7 isolates were subjected to the enterobacterial repetitive intergenic consensus (ERIC) polymerase chain reaction (PCR) typing to generate genetic fingerprints. The ERIC fragments were resolved by electrophoresis on 2% (*w*/*v*) agarose gels. The presence, absence and intensity of band data were obtained, exported to Microsoft Excel (Microsoft Office 2003) and used to generate a data matrix. The unweighted pair group method with arithmetic mean (UPGMA) and complete linkage algorithms were used to analyze the percentage of similarity and matrix data. Relationships between the various profiles and/or lanes were expressed as dendrograms. Data from groups of related lanes were compiled and reported on cluster tables. ERIC fragments ranged from one to 15 per isolate, and their sizes varied from 0.25 to 0.771 kb. A large proportion of the isolates produced an ERIC banding pattern with three duplets ranging in sizes from 0.408 to 0.628 kb. Eight major clusters (I–VIII) were identified. Overall, the remarkable similarities (72% to 91%) between the ERIC profiles for the isolate from animal species and their corresponding food products indicated some form of contamination, which may not exclude those at the level of the abattoirs. These results reveal that ERIC PCR analysis can be reliable in comparing the genetic profiles of *E.* *coli* O157:H7 from different sources in the North-West Province of South Africa.

## 1. Introduction

Shiga toxin-producing *Escherichia* *coli* (*E.* *coli*) (STEC) strains are pathogens that cause diseases in humans in many countries in the world [1,2]. Although there are more than 100 serotypes that are highly pathogenic to humans [3,4,5,6], serotype O157:H7 has been identified as the cause of most food and water-borne infections reported [7,8]. The diseases caused by *E.* *coli* O157:H7 include diarrhea, septicemia, bladder and kidney infections, pneumonia, neonatal meningitis and bacteremia in children and adults with AIDS, pyelonephritis, hemolytic uremic syndrome (HUS), hemorrhagic colitis (HC) and thrombotic thrombocytopenic purpura (TTP) [3,5,6,8,9,10]. These complications account for a high number of renal failures.

The pathogenicity of *E. coli* O157:H7 results from its ability to produce several virulence factors [11]. Generally, the Shiga toxins that are classified into *Stx1* and *Stx2* are considered to be the major virulence genes [12,13]. Unlike *Stx1*, other variants of *Stx2* have also been found to cause disease in both humans and animals [14,15]. There are other accessory virulence factors that mediate in the development of disease. These include the *eaeA* gene that codes for intimin, the *hlyA* gene and a host of others. Intimin facilitates intimate adherence of bacteria to intestinal epithelial cells, resulting in effacement of the surrounding microvilli. The pathogen is then able to exploit host cell signaling pathways to allow the colonization of their host [9].

*E.* *coli* O157:H7 infections usually result from the consumption of contaminated water and/or undercooked contaminated food products [16,17,18,19]. Cattle are considered as the principal host for these pathogens [20]. Despite this, the pathogen has also been isolated from several animal species that include pigs, sheep, deer, chicken and goats [21,22].

In developing countries, including South Africa, proper hygiene is not strictly implemented during the slaughtering of animals. Meat products that are contaminated during slaughter are potential sources for transmitting *E.* *coli* O157:H7 to humans [11,23] if consumed undercooked. It is important to implement proper hygiene in the farms, the abattoirs, the handling and/or the marketing of these food products to limit human infections.

Bacterial source tracking methods exist that determine the relationships of *E.* *coli* strains from different sources by comparing their genetic fingerprints [24,25]. In this study, we employed the enterobacterial repetitive intergenic consensus (ERIC) polymerase chain reaction (PCR) to amplify diverse regions of DNA that are flanked by conserved sequences to generate genetic fingerprints that are specific for *E. coli* O157:H7 isolates. Cross-contamination was assessed based on the similarities of the fingerprints of *E.* *coli* O157:H7 isolated from the different sources. The data obtained may be used in assessing the degree of risk posed to public health and for developing strategies to address *E.* *coli* O157:H7 infections.

## 2. Results and Discussion

### 2.1. Enterobacterial Repetitive Intergenic Consensus (ERIC) Polymerase Chain Reaction (PCR) Analysis

**Figure 1:**
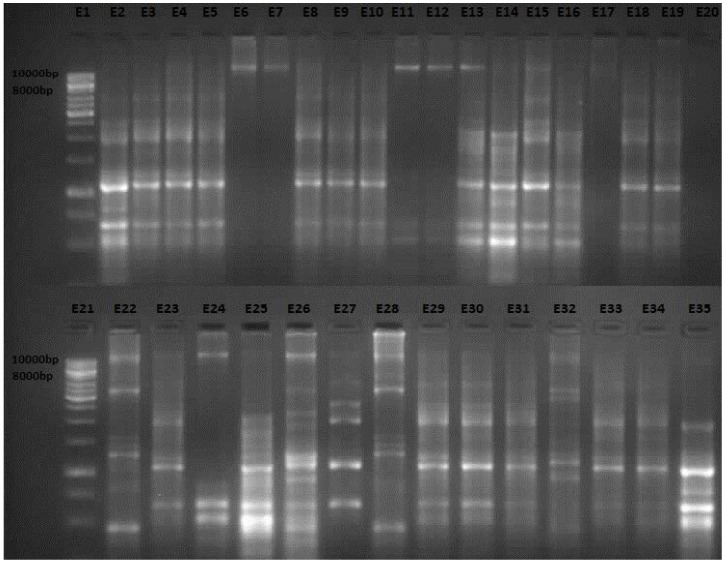
Enterobacterial repetitive intergenic consensus (ERIC) polymerase chain reaction(PCR) profiles of representative *Escherichia coli* (*E. coli*) O157:H7 isolates from the different sources. Lanes E1 and E21, 1 kb DNA ladder; Lanes E2–E6, isolates from water samples; Lanes E7–E13, isolates from cattle feces; Lanes E14–E20, isolates from beef; Lanes E22–E28, isolates from pig feces; Lanes E29–E34, isolates from pork samples; Lane E35, isolate from human stool samples.

A panel of 94 *E. coli* O157:H7 isolates from pigs, cattle, pork, beef, water and human stools were typed using the enterobacterial repetitive intergenic consensus ERIC PCR technique. Amplification reactions using primer ERIC2 produced DNA banding patterns that placed isolates into eight groups despite the source from which they were isolated. DNA fragments generated ranged from one to 15 per isolates per reaction, and the sizes varied from 0.25 to 0.771 kb (Figure 1). In general, a large proportion of the isolates produced a DNA banding pattern that had three duplets ranging in sizes from 0.408 to 0.628 kb. Visually, the ERIC patterns of *E. coli* O157:H7 isolates from water samples where similar to those from cattle feces, despite the differences in the sampling sites. Moreover, DNA fingerprints for isolates from cattle were also similar to those pork and human stool samples collected from supermarkets in the different cities.

**Figure 2:**
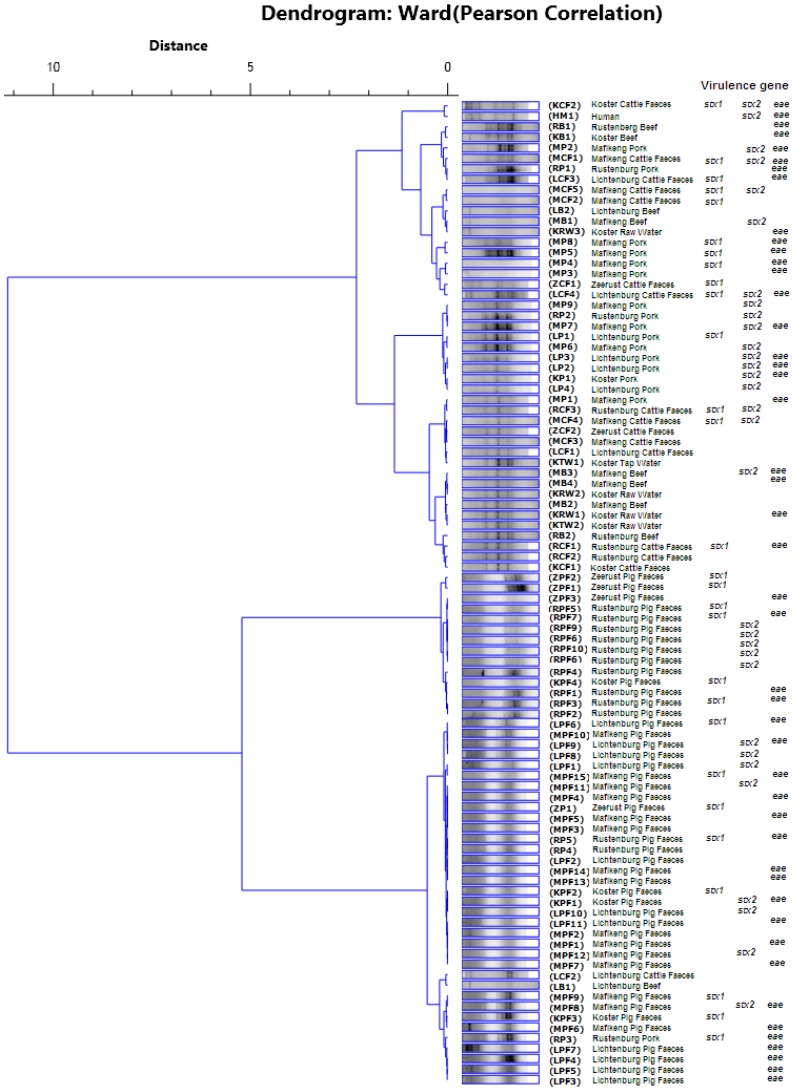
Dendrogram showing the relationship of *E. coli* O157:H7 isolated using the ERIC PCR analysis.

A comparison of the clustering patterns generated with the ERIC DNA profiles for all the 94 *E. coli* O157:H7 isolates revealed eight clusters (I–VIII). The largest cluster was cluster seven (VII) with 25.5% of the isolates typed (Figure 2). This cluster was dominated by isolates from feces samples from pigs in Mafikeng. Moreover, only isolates from pigs were found in this cluster. Similarly, clusters six (VI) and one (I) had only isolates from pigs, although it was dominated by those from the feces samples of animals in Rustenburg and pork in Lichtenburg and Mafikeng, respectively. A large proportion (80%) of the isolates from water in Koster were grouped in cluster two (II) together with isolates from cattle feces in Koster, Mafikeng, Lichtenburg and Rustenburg. However, their similarities with isolates from beef in these cities indicated that cross-contamination and the consumption of undercooked contaminated meat might have contributed to their presence in water. The *E. coli* O157:H7 isolate obtained from the human stool sample in Mafikeng was grouped in cluster three (III), which was the smallest in terms of the percentage representation of isolates. This human isolate had a similar ERIC profile with an isolate from cattle feces in Koster (Table 1).

Overall, the remarkable similarities (72% to 91%) between the ERIC profiles for the isolate from the different animals species and their corresponding food products as identified in clusters one (I), six (VI) and seven (VII) for pig isolates and clusters two (II) for isolates from cattle and water indicated that the ERIC DNA fingerprints was more effective in differentiating between isolates from different species (Table 1). The data obtained from the ERIC profiles of these *E. coli* O157:H7 isolates revealed that there is a need to reduce the level of contamination of meat products sold in supermarkets with intestinal contents (Cluster VII). However, it is also suggested that ERIC PCR proved to be very reliable in the typing of isolates from different species and, hence, could be of great importance in determining the source of *E. coli* O157:H7 contamination in the study area. In a preceding study [26], the virulence gene profiles of the isolates were determined and are indicated in Figure 2. Some of the isolates had been found to possess Shiga toxin genes and other putative virulence factors. *E. coli* O157:H7 isolates that possess the *eae* gene are highly associated with human disease [27], and there is usually a correlation between the *eae* gene and Shiga toxin genes [21,28]. A large proportion of the isolates used in the ERIC typing analysis possessed the *eae* gene, including the isolate from a human subject who was suffering from diarrhea. It is thus suggested that in the sampled area, direct contact with animals that shed *E. coli* O157:H7 in their feces should be controlled. This would reduce the transmission of these pathogens to humans.

### 2.2. Discussion

The objective of the present study was to determine the genetic relationship of *E. coli* O157:H7 isolated from diarrheal humans, pigs, cattle, beef, pork and water samples in the North-West Province of South Africa using genetic fingerprints generated from genomic DNA. A total of 94 randomly selected *E. coli* O157:H7 isolates from pigs, cattle, pork, beef, water and human stools were used. However, a limitation was the fact that only one isolate obtained from human stool was used for ERIC typing. Despite this, the study was designed to assess the commonness of *E. coli* O157:H7 isolates from animals species, their corresponding food products, water and humans. A motivation was the fact that in a previous study, *E. coli* O157:H7 from these sources had been reported to possess similar multiple antibiotic resistant phenotypes [29]. It was therefore suggested that improper farm management techniques, a lack of proper hygiene and the consumption of improperly cooked contaminated food products may account for the transmission of these pathogens to humans. In the present study, therefore, ERIC PCR analysis has been employed to amplify diverse regions of DNA that are flanked by conserved sequences to generate genetic fingerprints that are specific for particular isolates. Cross-contamination was assessed based on the similarities of the fingerprints of *E.* *coli* O157:H7 isolated from the different sources. The data obtained may be useful in assessing the health risk these contaminated food products and water posed to consumers in the area. Furthermore, these findings may assist in developing strategies to reduce *E.* *coli* O157:H7 infections in humans.

**Table 1:** Proportion of *Escherichia coli* (*E. coli*) O157:H7 from different species and/or sources with the various clusters based on the Enterobacterial Repetitive Intergenic Consensus (ERIC) Polymerase Chain Reaction (PCR) analysis. *N*, number of isolates with similar fingerprints/genetic profiles.

Specie/Source	Sample Type/Site	Cluster I *N* = 9	Cluster II *N* = 17	Cluster III *N* = 2	Cluster IV *N* = 6	Cluster V *N* = 11	Cluster VI *N* = 14	Cluster VII *N* = 24	Cluster VIII *N* = 11
Cattle	Mafikeng feces	0	2	0	1	2	0	0	0
	Mafikeng beef	0	3	0	0	1	0	0	0
	Lichtenburg feces	0	1	0	1	1	0	0	1
	Lichtenburg beef	0	0	0	0	1	0	0	1
	Koster feces	0	1	1	0	0	0	0	0
	Koster beef	0	0	0	1	0	0	0	0
	Zeerust feces	0	1	0	0	1	0	0	0
	Rustenburg feces	0	3	0	0	0	0	0	0
	Rustenburg beef	0	1	0	1	0	0	0	0
Pigs	Mafikeng feces	0	0	0	0	0	0	12	3
	Mafikeng pork	3	1	0	1	4	0	0	0
	Lichtenburg feces	0	0	0	0	0	0	7	4
	Lichtenburg pork	4	0	0	0	0	0	0	0
	Koster feces	0	0	0	0	0	1	2	1
	Koster pork	1	0	0	0	0	0	0	0
	Zeerust feces	0	0	0	0	0	3	0	0
	Zeerust pork	0	0	0	0	0	0	1	0
	Rustenburg feces	0	0	0	0	0	10	0	0
	Rustenburg pork	1	0	0	1	0	0	2	1
Humans	Mafikeng (feces)	0	0	1	0	0	0	0	0
Water	Koster (Tap)	0	2	0	0	0	0	0	0
	Koster (River)	0	2	0	0	1	0	0	0

Generally, ERIC PCR was able to distinguish among isolates from particular sampling sites and/or species. In most instances, it was able to show that isolates from a particular farm, food product obtained from supermarkets in particular city or water had similar ERIC profiles and clustered in the same group. It had been reported that the high degree of sequence similarity between bacterial isolates usually reflects descent from a common ancestor, and this explains their phylogenetic relatedness [30]. Moreover, *E.* *coli* O157 isolates from a particular geographic location with similar genetic and antibiotic resistant profiles had been reported to be related genetically [31]. In both instances the isolates clustered in the same similarity group [30,31]. ERIC PCR revealed that the isolates screened in the present study had a wide range of genetic diversities and the method was very sensitive in detecting slight differences between isolates from different species. The major implication of the finding is that the ERIC PCR analysis could serve as a more effective tool in the routine surveillance of *E.* *coli* O157:H7 in the area.

## 3. Experimental Section

### 3.1. Sample Collection

One hundred fecal samples were collected from cattle, pigs and humans, while 40 water samples were collected, each from taps and river catchments within the North-West Province of South Africa. Meat samples comprised 40 pork and 40 beef samples bought from supermarkets in some major cities in the province. The meat samples were placed in sterile plastic bags and labelled based on sample type and the area of collection. Human fecal samples were collected from 20 patients that visit the Mafikeng provincial hospital for cases of diarrhea. The hospital does not perform routine screening for *E. coli* O157:H7 and, as such, the impact of this pathogen in diarrheal cases within the area is unknown. The isolation of *E.* *coli* O157:H7 from human stool samples was performed at the microbiology laboratory of the Mafikeng Provincial hospital. The samples were handled with care, and all ethical procedures were enforced during the isolation of *E. coli* O157:H7. They were obtained without any indication of patient identity, used only for bacterial isolation and properly disposed of by the laboratory staff of the hospital immediately after analysis. Animal samples were collected directly from the rectum of animals using sterile arm-length gloves and were placed in sterile sample collection bottles. Water samples were collected in 100 mL collection bottles. The meat, feces and water samples were immediately transferred on ice to the laboratory for analysis. Upon arrival in the laboratory, all the samples were analyzed immediately or held at 4 °C for not more than 48 h before analysis. Table 2 indicates the numbers of the different samples collected from the stations sampled.

#### 3.2. Isolation of E. coli O157:H7

##### 3.2.1. Human Stool and Animal Fecal Samples

Two grams of fecal samples were dissolved in 5 mL of modified trypticase soy broth (Merck Diagnostics, Hertfordshire, UK), supplemented with novobiocin (2 µg/mL) and cefixime (50 ng/mL). The broth was incubated at 37 °C for 24 h [32]. Ten-fold serial dilutions of the pre-enriched samples were performed using 2% peptone water. Aliquots of 100 µL from each dilution were plated onto sorbitol-MacConkey agar (SMAC) supplemented with cefixime (50 ng/mL) and potassium tellurite (25 mg/mL). The plates were incubated at 37 °C for 24 h [32].

**Table 2:** Area of collection, source, nature and number of samples collected during the study.

Sample Source	Sampling Area	Nature of Sample	Number of Samples
Pigs	Koster	Fecal sample	8
	Lichtenburg	Fecal sample	8
	Mafikeng	Fecal sample	8
	Rustenburg	Fecal sample	8
	Zeerust	Fecal sample	8
Pigs	Koster	Pork	8
	Lichtenburg	Pork	8
	Mafikeng	Pork	8
	Rustenburg	Pork	8
	Zeerust	Pork	8
Bovine	Koster	Fecal sample	8
	Lichtenburg	Fecal sample	8
	Mafikeng	Fecal sample	8
	Rustenburg	Fecal sample	8
	Zeerust	Fecal sample	8
Bovine	Koster	Beef	8
	Lichtenburg	Beef	8
	Mafikeng	Beef	8
	Rustenburg	Beef	8
	Zeerust	Beef	8
Water	Koster	Water	8
	Lichtenburg	Water	8
	Mafikeng	Water	8
	Rustenburg	Water	8
	Zeerust	Water	8
Human	Mafikeng Provincial Hospital	Fecal sample	20

#### 3.2.2. Meat Samples

For the isolation of *E. coli* O157:H7, 2 g of beef or pork obtained from supermarkets in some major cities in the North West Province, South Africa (Table 2) were washed in 5 mL of modified trypticase soy broth (Merck Diagnostics), supplemented with novobiocin (2 µg/mL) and cefixime (50 ng/mL). The broth was incubated at 37 °C for 24 h [32]. Ten-fold serial dilutions of the pre-enriched samples were performed using 2% peptone water. Aliquots of 100 µL from each dilution were plated onto sorbitol-MacConkey agar (SMAC) supplemented with cefixime (50 ng/mL) and potassium tellurite (25 mg/mL). The plates were incubated at 37 °C for 24 h [32].

#### 3.2.3. Water Samples

Five hundred milliliters of water were collected from each source per collection. Aliquots of 100 mL from each of the sample were filtered through 0.45-µm grid filter units (Type HA) using a Gelman Little Giant pressure/vacuum pump machine (model 13156; Gelman Sciences, Ann Arbor, MI, USA). The filters were placed on sorbitol-MacConkey agar (SMAC) supplemented with cefixime (50 ng/mL) and potassium tellurite (25 mg/mL). The plates were incubated at 37 °C for 24 h [7].

Presumptive *E. coli* O157:H7 colonies were colorless on CT-sorbitol-MacConkey agar, and fifty six of these from each sample were sub-cultured onto CT-sorbitol-MacConkey agar. The plates were incubated at 37 °C for 24 h [32]. The isolates were preserved by culturing on nutrient agar, and the plates were incubated at 37 °C for 24 h. The plates were stored at room temperature until the isolates were genotypically characterized by ERIC PCR analysis.

### 3.3. E. coli Control Strains

*E. coli* O157:H7 (ATCC 43889) and *E. coli* O157:H7 (NCTC 12900) were used as positive control strains during the isolation and identification of isolates.

### 3.4. Extraction of Genomic DNA

Genomic DNA was extracted from the presumptive *E. coli* O157:H7 isolates using the alkaline lysis method [33]. DNA extracted from *E. coli* O157:H7 isolates and control strains were quantified by measuring the absorbance at 260 nm using a UV-visible spectrophotometer (model S-22, Boeco, Hamburg, Germany).

### 3.5. Molecular Identification of E. coli O157:H7 Isolates

The identities of the suspected isolates were confirmed using the amplification of the *rfb*O_157_ and the *fliC*_H7_ gene fragments [26]. Moreover, an evaluation of the virulent gene combinations of the isolates was also performed through amplification of the *stx_1_*, *stx_2_*, *eae* and *hlyA* gene fragments [26], and details of the various virulence gene combinations for the isolates are shown in Figure 2. A total of 94 *E. coli* O157 strains from different sources were subjected to ERIC PCR typing to determine their commonness and genetic relationships. The makeup of this is shown in Table 3.

**Table 3:** Number of *E.* *coli* O157:H7 isolates from the different species and/or sources that were used for genotypic typing. NT, not tested.

Source	Humans	Pigs		Cattle		Water		Total
	Feces	Feces	Pork	Feces	Beef	Taps	River Catchment	
Mafikeng	1	15	9	5	4	0	0	35
Lichtenburg	NT	11	4	4	2	0	0	22
Koster	NT	4	2	2	1	2	3	14
Rustenburg	NT	10	5	3	2	0	0	22
Zeerust	NT	3	1	2	0	0	0	2
Total	1	43	20	16	9	2	3	94

### 3.6. ERIC PCR Assays

To perform the ERIC PCR analysis, a Peltier Thermal Cycler (model PTC-220 DYAD™ DNA Engine, Bio-Rad, Hercules, CA, USA) was used for the PCR amplifications. The reactions were performed in 25 µL volumes that included 50 ng of template DNA, 50 pmol of the ERIC2 primer (5'-AAGTAAGTGACTGGGGTGAGCG-3'), 1× Master mix 0.4 mM of each dNTP, 0.05 U/µL *Taq* DNA polymerase, 4 mM MgCl_2_, 1× PCR reaction buffer and nuclease-free water. All the PCR reagents were Fermentas (Pittsburg, PA, USA), products and supplied by Inqaba Biotec, Pretoria, South Africa. The PCR cycling conditions involved an initial denaturation step of 95 °C for 2 min, 30 cycles of 94 °C for 3 s, 50 °C for 1 min, 65 °C for 8 min and a final elongation step at 65 °C for 8 min. The PCR products were held at 4 °C until electrophoresis.

### 3.7. Agarose Gel Electrophoresis

The PCR products were separated by electrophoresis on a 1% (*w*/*v*) agarose gel. The gels were stained in ethidium bromide (0.001 µg/mL) for 15 min, and the amplicons were visualized under UV light [33]. A Gene Genius Bio Imaging System (Syngene, Synoptics, Cambridge, UK) was used to capture the image using GeneSnap (version 6.00.22) software. Images were analyzed using GeneTools (version 3.07.01) software (Syngene, Synoptics) to determine the relative sizes of the amplicons, and the images were saved as tif image files.

### 3.8. Statistical Analysis

The fingerprints were compared and analyzed with the TotalLab Phoretix 1D Pro software (TotalLab Ltd., Newcastle, UK). The presence, absence and intensity of band data were obtained, exported to Microsoft Excel (Microsoft Office 2003) and used to generate a data matrix. The unweighted pair group method with arithmetic mean (UPGMA) and complete linkage algorithms were used to analyze the percentage similarity and matrix data. Relationships between the various profiles and/or lanes were expressed as dendrograms. Data from groups of related lanes were compiled and reported on cluster tables.

## 4. Conclusions

ERIC PCR revealed that the isolates screened in the present study had a wide range of genetic diversities, and the method was very sensitive in detecting slight differences between isolates from different species. The major implication of the finding is that the ERIC PCR analysis could serve as a more effective tool in the routine surveillance of *E.* *coli* O157:H7 in the area.
